# Comparison of Continuous Epidural Infusion of Bupivacaine and Fentanyl Versus Patient Controlled Analgesia Techniques for Labor Analgesia: A Randomized Controlled Trial (RCT)

**Published:** 2020

**Authors:** Raha Khaneshi, Sousan Rasooli, Farnaz Moslemi, Sanam Fakour

**Affiliations:** 1- Department of Anesthesiology, Alzahra Hospital, Faculty of Medicine, Tabriz University of Medical Sciences, Tabriz, Iran; 2- Department of Cardiothoracic Surgery, Alzahra Hospital, Faculty of Medicine, Tabriz University of Medical Sciences, Tabriz, Iran

**Keywords:** Bupivacaine, Epidural analgesia, Fentanyl, Labor pain

## Abstract

**Background:**

To diminish labor pain, several techniques have been used in developed countries. In the current randomized controlled trial, the use of epidural analgesia via PCEA pump with and without background infusion of analgesic was studied.

**Methods:**

In this double-blinded controlled trial, 60 women were enrolled and randomly assigned to study groups for receiving epidural analgesia during labor. All patients received initial bullous dose including 125 *mg* bupivacaine and 3 *mg/ml* fentanyl, and the first group patient (CI) received background infusion of 8 *ml/hr* and the second group (PCEA) received 10 *ml* bullous dose of 125 *mg* bupivacaine combined with 100 *mcg* fentanyl (2 *ml*) via epidural catheter. The Visual Analogue Scale (VAS) of 0–10 was measured 20 *min* after drug injection. The chi-square and student T-test were used for comparing variables between groups, and 0.05 was considered as the level of significance.

**Results:**

There was no significant difference in terms of demographic variables. Mean duration of the second stage of labor was significantly lower in patients received continuous infusion (CI) (p<0.0001). However, the total administered fentanyl dose was significantly higher in patients who underwent PCEA (p<0.0001). Besides, the CI group had a significantly lower rate of patient-controlled injection compared to PCEA patients (p<0.0001). However, there was no significant difference between patients’ satisfaction and VAS in study groups.

**Conclusion:**

Epidural analgesia using PCEA combined with continuous infusion did not provide higher analgesia or patients’ satisfaction compared to PCEA alone; however, it led to a decreased rate of drug injection and total administered dosage.

## Introduction

Labor is a physiologic process that is associated with severe pain (1). Wall and Melzark described labor pain control and its mechanisms with the goal of painless labor concerning controlled fetal and maternal complications (2). Epidural analgesia provides a safe approach during labor that provides adequate analgesia without motor block (3). Despite its advantages, low dose local analgesic leads to delayed analgesia and pain control. Opioids such as fentanyl are suggested to shorten the analgesia duration and onset in bullous doses (4, 5). Bupivacaine is a local anesthetic that provides adequate analgesia in association with long durability, the sensory block without motor disturbances, and decreased prevalence of tachyphylaxis (2). In addition, low placental transfer prevents fetal complications during labor compared to other local anesthetics.

With due attention to variety of drugs and analgesia methods introduced to control labor pain, there is a controversy over the most effective pain management method. Patient-controlled epidural analgesia (PCEA) is a unique analgesia method first introduced by Gambling in obstetrics that provides several advantages when compared to continuous epidural infusion (CEI) technique; the benefits include more efficient analgesia, reduction in dose of administered local anesthetics, decreased rate of adverse events and reduced lower extremity motor block (6–8). Besides, self-administered epidural drug and subsequent pain control provide significant psychological advantages during labor (9, 10). Although the association of PCEA with higher maternal satisfaction is unclear, which may be due to unstandardized measuring tools, it turned out to be a popular technique based on its efficacy (11). Several studies have been carried out to compare PCEA and CEI in terms of adequate analgesia in women undergoing labor, but the best analgesia technique is still controversial. However, some studies demonstrated that PCEA might cause acute pain in patients and extra analgesics administration might be needed (12).

On the other hand, CEI leads to insufficient pain control and increased rate of analgesics side effects (13). There are two PCEA regimens, including demand only PCEA and PCEA with continuous background infusion. Although PCEA plus continuous background infusion efficacy in labor is controversial, demand only PCEA has been suggested to provide low pain intensity without any effect on analgesia. Considering the discrepancies mentioned above, an attempt was made to evaluate CEI efficacy in labor compared with demand-only PCEA.

## Methods

### Patients:

Study sample size was calculated with due attention to the previous study by Srivastava et al. (14). Considering the 18% difference of decrease in pain intensity between study groups, error rate of 0.05 and study power of 80%, based on allowable difference between blood glucose levels, case and control groups contained 30 patients, which increased to 34 patients in each group to eliminate the risk of sample loss. Therefore, 68 women were candidates to undergo delivery, selected via simple sampling.

### Procedure:

**Figure 1.**
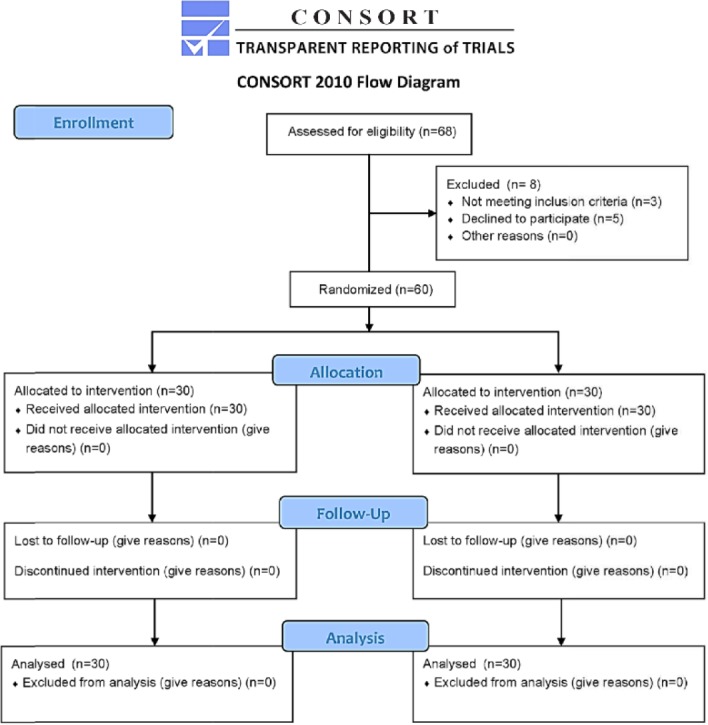
The CONSORT flow diagram of the randomized clinical trial

A controlled randomized trial was carried out between May 2015 and November 2015, at Alzahra women’s hospital, Tabriz University of Medical Sciences. The study was approved by the ethics committee of the Vice Chancellor of Research and Development, Tabriz University of Medical Sciences (Approval No: TBZMED.REC. 1394.1046), and registered in Iranian Registry of Clinical Trials (Primary registry in the WHO registry network) (www.irct.com, ID No: IRCT2014 121610765N6). All pregnant women aged between 18 to 40 years were candidates to undergo vaginal delivery with vertex presentation and were classified as class I and II patients according to American Society of Anesthesiology Classification System. Pregnant women who were class III cases based on ASA or higher or had a history of cardiopulmonary disorders, diabetes mellitus, contraindications for epi dural analgesia, coagulopathies, fetal presentations other than vertex, were excluded, as well as patients with a history of chronic pains or analgesic consumption. All patients provided written, informed consent to participate in the study. Sixty patients were randomly assigned to study groups as follows: (A) epidural analgesia provided via continuous epidural infusion technique, and (B) patients received PCEA technique for epidural analgesia (Figure 1). Randomization was performed using a computer via RandList Software.

All patients’ demographic information, parity, gestational age, amniotic sac status, and fetal heart rate were recorded, as well as vital signs, including blood pressure, heart rate, arterial oxygen saturation. Epidural analgesia maintained throughout the first stage of labor when the cervix dilatation was 3–4 *cm*. If progress during the second stage of labor was inadequate after an hour, the infusion was discontinued. While patients were in sitting position, puncture was performed in L_3_–L_4_ inter–space and epidural catheter was implemented. Test dose contained 3–4 *ml* of 1.5% lidocaine combined with 1/200000 epinephrine to evaluate the function of the catheter. The Turen pump, continuous and bullous type, with a lockout time of 15 minutes was used for epidural infusion. All patients received a loading dose of 10 *ml* solution that included 125 *mg* of 0.5% bupivacaine combined with 2 *ml* of 3 *μg/ml* fentanyl. Afterward, 8 *ml* of the loading solution was infused each hour to obtain maintenance dosage in the first group (CEI: 30 patients). However, in PCEA group (30 patients), infusion pump contained 125 *mg* of 0.5% bupivacaine with 2 *ml* of 100 *μg* fentanyl in 100 *ml* of normal saline, and in case of demand for analgesics, patients received 2 *ml* of the solution by pushing the pump button. However, in case of inadequate analgesia, anesthesiologist administered 8 *ml* of the solution via the epidural catheter. Also, if there was intransient fetal bradycardia or impaired non-stress test (NST), analgesic infusion was discontinued temporarily and further consultation was requested from obstetricians. A blinded anesthesiologist recorded the pain intensity 20 *min* after drug administration and then hourly using Visual Analogue Scale (VAS) of 0–10, that 0 showed no pain and 10 indicated the worst pain. Neonatal APGAR was calculated in the first and 5th minutes. Adverse effects of the epidural analgesia techniques such as lower extremity weakness, hypotension, drowsiness, altered O_2_ saturation, nausea and vomiting, and pruritus were evaluated and further treatment was applied.

### Statistical analysis:

The chi-square test was used for comparing two qualitative variables in each time, and student t-test for comparing quantitative variables between groups. The level of significance was set at 0.05, and all results were expressed by frequency (Percentage) for qualitative variables and Mean±SE for quantitative variables. Repeated measure test was used to compare means between independent groups by SPSS 15 Software.

## Results

Of sixty-eight patients, eight patients were excluded, whereas five patients quit the study at will, and three patients underwent cesarean section due to different reasons. Patients’ demographic data is shown in table 1. There was no significant difference between study groups in terms of age, body weight, gestational age, parity, and cervix dilatation during analgesia administration. Among sixty patients, 26 (43.3%) and 34 (56.7%) mothers were primigravida and multigravida, respectively.

**Table 1.** Non-continuous variables including patients’ pre-intervention data and number of the injections during labor in the study groups

	**Minimum**	**Maximum**	**Median**	**p**
**Parity number**				
PCEA ^*^	1	3	2	0.13
CEI ^**^	1	3	1	
**Cervix dilatation (*cm*)**				
PCEA	3	4	4	0.07
CEI	3	5	4	
**Injection times**				
Physician				
PCEA	0	2	0	0.1
CEI	0	2	0	
Patient				
PCEA	6	23	9	<0.001
CEI	0	5	0	

* Patient-Controlled Epidural Analgesia

** Continuous Epidural Infusion

Mean durations of labor at second stage were 114±26.98 *min* and 73.33±13.97 *min* in PCEA and CEI groups, respectively, that showed significant lower duration in patients received CEI analgesia technique (p<0.0001). Newborn APGAR scores were calculated during the first and 5th *min* after birth and mean and mode of the APGAR scores considering the study group are listed in table 2. Comparing newborns APGAR scores with regard to the analgesia technique provided for mothers, there were significant difference in terms of the 1st *min* APGAR scores, which showed a higher APGAR score in newborns in comparison to the scores obtained via CEI analgesia technique (p= 0.046); however, there was no difference between newborn APGAR scores in the 5th *min* (p=0.078). Lower extremity motor block level was defined via Modified Bromage Scale (MBS) which revealed similar results between study groups. The total dosage of administered drugs is listed in table 2. Comparing the mean amount of administered bupivacaine, no significant difference was observed (p=0.097). However, the mean amount of fentanyl was significantly higher in patients who underwent PCEA technique (p<0.0001).

**Table 2.** Continuous variables including APGAR scores, injection dosage and patients’ satisfaction scores

	**Mean±SD**		**p**

	**PCEA**	**CEI**	
**APGAR score**			
1st *min*	8.53±1.1	9.03±0.76	0.046
5th *min*	10	9.90±0.30	0.078
**Drug dosage**			
Bupivacaine (*ml*)	37.06±0.37	34±6.69	0.09
Fentanyl (*mcg*)	61.23±12.53	49.83±12.6	<0.001
**Patients’ satisfaction**	84.33±19.24	83±20.86	0.79

Patients for whom drug was prescribed by anesthesiologist were compared in groups. Majority of patients had no demand for extra administration by the physician in both study groups (PCEA: 70%, CEI: 86.6%) and there was no significant difference between the study groups (p=0.105). Comparing patients satisfaction considering study group, the mean satisfaction score was 84.33± 1924 in the PCEA group and 83±20.85 in the CEI group which revealed no significant difference between groups (p=0.798).

In order to assess blood pressure changes subsequent to epidural analgesia, mean arterial pressure was calculated for patients before analgesia induction, 5 *min* later, 20 *min* later and at the end of the labor. Mean arterial pressure in each period is listed in table 3. Comparing blood pressure alteration during measurement periods via repeated measure test showed that patients who received CEI analgesia were more susceptible to blood pressure alteration compared to PCEA group (p< 0.0001).

**Table 3.** Average of the mean arterial pressure (MAP) in different periods in the study groups

**Group**	**Mean arterial pressure (MAP)**				**p**

	**Primary**	**5 *min* after analgesia**	**20 *min* after analgesia**	**End of the labor**	
**PCEA**	95.67±9.65	93.23±7.94	95.97±6.81	96.57±14.79	<0.001
**CEI**	101.70±6.59	94.93±8.14	90.13±8.14	94.40±7.57	

In addition, pain intensity measured in all patients during different periods via VAS is shown in table 4. In order to prevent any bias, pain intensity was compared before analgesia induction, and no significant difference was observed between patients of the study groups (p=0.129). Comparing pain intensity changes between study groups among different periods, although pain intensity decreased in both groups following epidural analgesia administration, PCEA group had more severe alteration rather than CEI group (p<0.0001), which reveals higher efficacy of CEI analgesia technique.

**Table 4.** Median of the pain severity according to the VAS in the study groups

**Group**	**VAS (Median (min-max))**						**p**

	**Base**	**1st *hr***	**2nd *hr***	**3rd *hr***	**4th *hr***	**5th *hr***	
**PCEA**	6 (5–8)	0 (0–4)	0 (0–4)	0 (0–4)	0 (0–3)	0 (0)	<0.001
**CEI**	5.5 (5.6)	0 (0–3)	0 (0–4)	0 (0–4)	0 (0–4)	0 (0)	

Pruritus was the only adverse effect reported in both groups as follows: 17 (56.6%) patients of CEI group and 15 (50%) patients of PCEA group needed further treatment. However, the analysis showed no difference in terms of pruritus prevalence between study groups (p=0.398).

## Discussion

The aim of taking analgesia during labor is to acquire adequate analgesia in association with the least maternal and fetal complications (5). Epidural analgesia has turned out to be a popular and widely accepted analgesia technique considering its several advantages, including demanded analgesia during primary stages of the labor (15). Patient-controlled epidural analgesia (PCEA) is an epidural analgesia technique that results in decreased demand for drug administration by the physician and less consumption of overall analgesic and opioid compared to other techniques such as CEI. PCEA includes two techniques of demand only PCEA and PCEA associated with background infusion; however, the overall benefits of techniques compared to each other is still controversial (9, 16). Although the use of epidural analgesia is well-established in clinical practice and its benefits are well described, similar to any other intervention, it can lead to some complications after the intervention (17). Severe complications, such as hematoma and infections, are infrequent, but they result in permanent disability in most of the cases (18). Thus, after the intervention, patients should be observed meticulously to evaluate potential sources of the complications (19). Besides, any alteration during neurologic examinations should be taken into consideration promptly to determine the underlying reasons for the deficits.

In the present double-blinded randomized controlled trial, pregnant women were divided into PCEA and CEI groups for epidural analgesia during labor. Our study results showed that PCEA technique led to prolonged duration of labor at second stage compared to the CEI group. Although newborns’ mean APGAR scores during the 1st *min* was lower in the PCEA group, there was no significant difference while comparing the 5th *min* APGAR scores. Overall, the comparison of drug consumption revealed a higher dose of fentanyl administration in the PCEA group, which may be in association with decreased 1st *min* APGAR scores in the PCEA group due to a higher dosage of opioid consumption. Considering blood pressure alteration in study groups, CEI group was more susceptible to mean arterial pressure changes in comparison to the CEI group. Although there was no significant difference in terms of patients’ satisfaction rate between study groups, CEI technique resulted in a more significant decrease in pain intensity compared to PCEA.

In a meta-analysis over the efficacy of PCEA with and without background infusion, patients received PCEA associated with background infusion had a lower rate of demand for further intervention to acquire demanded pain control. In a study by Bremerich et al., ropivacaine administration in combination with sufentanil resulted in better pain control and decreased the dose of drugs, when applied via PCEA in association with background infusion, compared to PCEA demand only technique (20). Similarly, in our study, although there was no significant difference in terms of pain intensity between PCEA and CEI techniques, PCEA technique led to an increased dosage in overall drug consumption, which emphasizes lower accuracy of demandonly PCEA. However, Okutomi et al. demonstrated that patients who received background infusion added to PCEA had a lower rate of demand for analgesic administration, but required higher drug dosage (21). Despite the findings of Okutomi study, in this study, it has been shown that the CEI technique is associated with decreased drug infusion without any difference in the rate of patients’ demand for analgesic application by the physician. The recent discrepancy between results may be due to a different rate of drug infusion applied.

Halpern et al. compared ropivacaine and bupivacaine drugs in PCEA with background infusion and demand only PCEA during labor (22). They concluded that background infusion associated with PCEA provides better epidural analgesia during labor without lower extremity motor block. While, in our study, no significant difference was reported in terms of patients’ satisfaction and pain control, there was a severe reduction in pain intensity in patients received CEI technique. However, in none of the study groups, lower extremity motor block was reported, and pruritus was the only adverse effect that needed no treatment in any of the patients. Srivastava et al. showed no significant difference in terms of patients satisfaction, pain intensity, and newborn APGAR scores while comparing PCEA with CEI techniques (14).

Likewise, in our study, comparing patients’ satisfaction and pain intensity, no significant difference was found between study groups; however, the 1st *min* APGAR scores had a reduction in the PCEA group. Besides, the overall drug dosage administered in the PCEA group was higher than the CEI technique. Therefore, it is hypothesized that a higher dosage of analgesic administration in the PCEA group resulted in decreased APGAR scores in newborns born from mothers who received demand only PCEA technique for labor. On the other hand, the discrepancy between our results and that of Srivastava et al. in terms of APGAR scores and higher dosage of infused drugs can be based on higher infusion rate (10 *ml/hr*) administered in Srivastava’s study when compared to our infusion rate in CEI groups (8 *ml/hr*). In another study by Vallejo et al., they reported that although demand only PCEA is not associated with better pain control and decreased pain intensity compared to CEI technique during labor, it decreases the dosage of analgesic infusion (23).

On the contrary, our results showed increased analgesic consumption in the PCEA demand only group compared to CEI technique. This difference between results emphasizes the importance of patient education in the use of infusion pump through demand only PCEA technique, that leads to high consumption of analgesic in PCEA group in our study. Despite our hypothesis that suggested better analgesia and patients’ satisfaction in demand only PCEA technique, our results revealed that demand only PCEA neither provides better pain control, nor decreases pain intensity, rather it increases the dosage of administered analgesic and leads to lower APGAR scores in newborns of the PCEA administered mothers.

## Conclusion

Demand only PCEA technique does not provide better pain control and patients satisfaction compared to CEI analgesia technique and increases the dosage of administered analgesic.
